# A SYSTEM TO IDENTIFY INHIBITORS OF MTOR SIGNALING USING HIGH-RESOLUTION GROWTH ANALYSIS IN S. CEREVISIAE

**DOI:** 10.1093/geroni/igz038.381

**Published:** 2019-11-08

**Authors:** Michael Kiflezghi, Matt Kaeberlein

**Affiliations:** University of Washington, Seattle, Washington, United States

## Abstract

Age is the main risk factor for cancer, cardiovascular disease, neurodegeneration and other diseases prevalent in the world’s aging population. These diseases increase the pain and suffering and result in billions of dollars in healthcare costs. Addressing the common risk factor may allow for simultaneous amelioration of these diseases providing personal and economic relief to people and societies around the globe. The mTOR signaling pathway has been shown to be a robust target in the aging process with its inhibition resulting in increased lifespan in model organisms from yeast to mice. Rapamycin is an FDA approved drug for use in transplant patients and is a potent and specific inhibitor of mTOR complex 1. Rapamycin’s use as an anti-aging therapeutic in otherwise healthy individuals is complicated by the occurrence of side effects. As such, groups studying inhibition of the mTOR pathway are searching for alternative inhibitors that may be able decouple the deleterious effects of rapamycin administration from the lifespan extending effects. To address this issue, we have developed a high-throughput yeast-based assay for the identification of novel mTOR inhibitors. By utilizing mutant strains with differential sensitivity to mTOR inhibition, comparative growth kinetics of microcultures exposed to an inhibitor can be used to discern the mTOR inhibitory status of a compound. Furthermore, the assay can provide mechanistic insight on a compound’s mode of inhibition providing a rich, fast readout of a compound’s potential for inhibiting mTOR. This approach allows for the screening of large libraries of compounds speeding the discovery process.

